# Scleromyxoedema and Thyroid Disease: A Case Report

**DOI:** 10.7759/cureus.90407

**Published:** 2025-08-18

**Authors:** Hajar El Hassani Taib, Syrine Hamada, Meryem El Moustaoui, Najoua Ammar, Nadia Ismaili, Laila Benzekri, Mariame Meziane

**Affiliations:** 1 Dermatology Department, Ibn Sina University Hospital Center, Mohammed V University, Rabat, MAR

**Keywords:** cholangitis, gammopathy, hashimoto, hypothyroidism, immunoglobulin, monoclonal, papular mucinosis, scleromyxoedema, thyroid

## Abstract

Scleromyxoedema (SM) is a rare cutaneous mucinosis characterised by waxy papules, diffuse skin induration, and systemic involvement such as gastrointestinal, cardiopulmonary, or neurological complications. The disease is strongly linked to the presence of monoclonal gammopathy. Traditionally, thyroid dysfunction is considered an exclusion criterion for the diagnosis of SM; however, clinical observations suggest that this association may occur in certain cases. We present the case of a 70-year-old woman with a history of hypothyroidism on levothyroxine for three months, who presented with progressive cutaneous sclerosis affecting the face, trunk, and limbs, along with dysphagia for solid food. Clinical examination revealed generalised induration of the skin and limited mouth opening. Skin biopsy confirmed the diagnosis of SM, revealing fibroblast proliferation and mucin deposition. Laboratory tests identified a monoclonal immunoglobulin G kappa gammopathy, elevated free kappa light chains, and persistent hypothyroidism with high levels of anti-thyroid peroxidase antibodies, which confirmed the diagnosis of Hashimoto’s thyroiditis. Liver abnormalities and biopsy findings were consistent with primary biliary cholangitis. The patient was treated with systemic corticosteroids and intravenous immunoglobulin (IVIG), but no clinical improvement was observed. The disease progressed, and she died five months after the last treatment cycle. This case illustrates the rare coexistence of SM and autoimmune thyroid disease, raising questions about whether thyroid dysfunction should systematically exclude SM diagnosis. The differential diagnosis with hypothyroid myxedema and systemic sclerosis is crucial, as their histopathological features differ significantly. Although IVIG is first-line therapy for SM and achieves good response rates in many cases, therapeutic failure, as seen here, highlights the need for further research into optimal treatment strategies for SM with overlapping autoimmune conditions.

## Introduction

Scleromyxoedema (SM), or papulosclerotic mucinosis, is characterized by the accumulation of mucin in the dermis in patients with a monoclonal gammopathy. This condition belongs to the group of primary, or specific, cutaneous mucinoses, which differ from secondary cutaneous mucinoses, in which mucin deposition is merely an incidental histological finding within another dermatosis. SM is an exceptionally rare disease, with unknown incidence or prevalence, most often reported as isolated cases or in small case series. It is characterized by the presence of waxy papules overlying diffuse skin induration in patients with monoclonal gammopathy, typically affecting adults with a mean age of 51.5 years and showing a female predominance [1].

SM is a progressive disease of unknown etiology, associated with systemic manifestations involving the gastrointestinal, pulmonary, musculoskeletal, endocrine, articular, and neurological systems, which may lead to significant morbidity and a mortality rate of around 30-40% within two years [2,3]. According to the classification of lichen myxedematosus proposed by Rongioletti and Rebora in 2001, the diagnosis of SM relies on a combination of clinical, histopathological, and laboratory features. Specifically, it requires a generalized papular and sclerodermoid eruption, histopathological evidence of mucin deposition in the reticular dermis with fibroblast proliferation and fibrosis, and the presence of serum monoclonal gammopathy. Thyroid dysfunction is considered an exclusion criterion, indicating that patients with thyroid disease are generally not classified as having SM [4].

We report here a rare case of SM associated with Hashimoto’s thyroiditis.

## Case presentation

A 70-year-old woman suffered from cutaneous sclerosis for six months, progressively affecting the face, trunk, upper limb, and lower limb. The evolution was marked two months ago by the worsening of cutaneous sclerosis with dysphagia to solids, all evolving in a context of asthenia and apyrexia. Furthermore, she did not present with dyspnea, arthralgia, or Raynaud's phenomenon. She had a medical history of hypothyroidism, diagnosed three months ago, and was on levothyroxine sodium.

**Figure 1:**
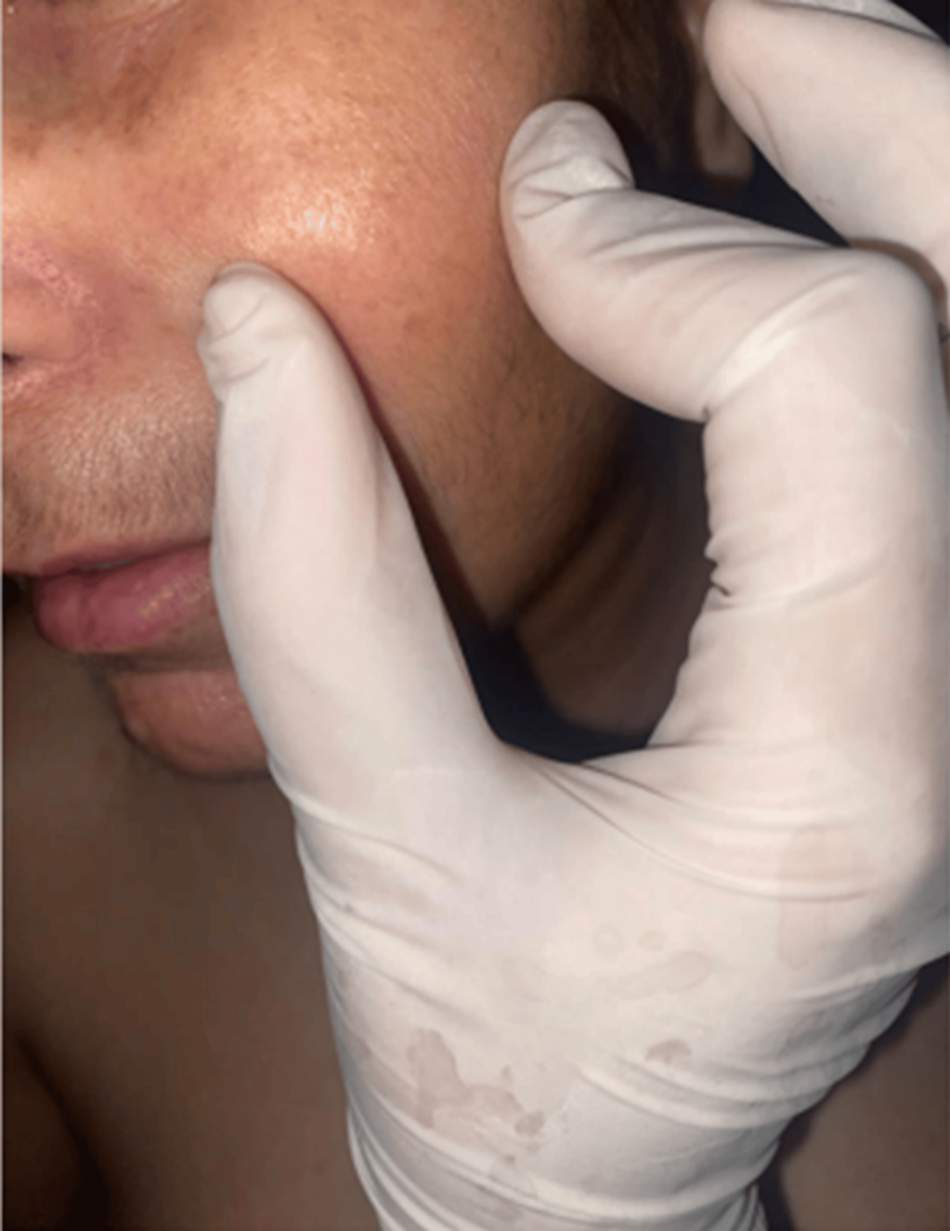
Cutaneous sclerosis of the cheek

**Figure 2:**
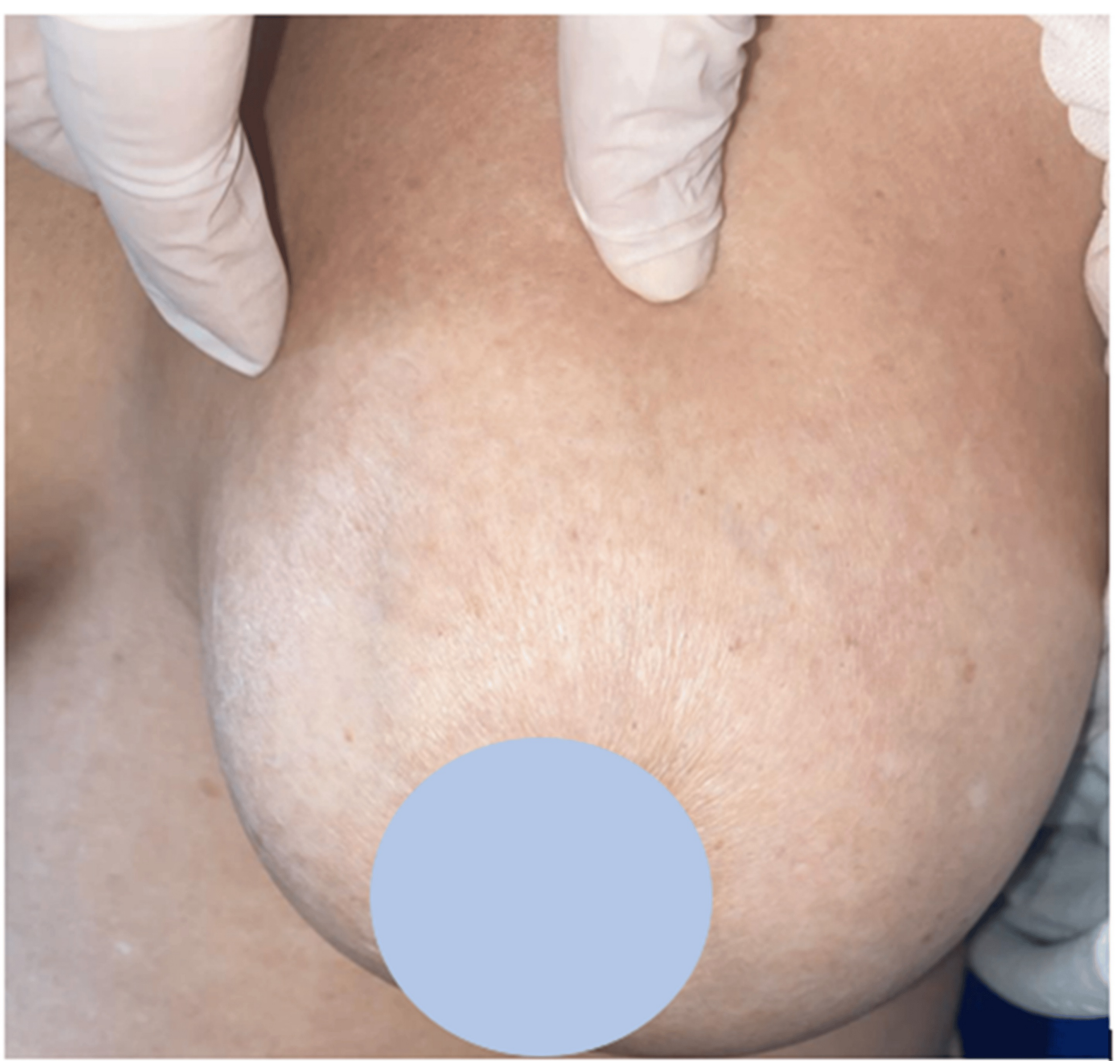
Cutaneous sclerosis of the breast

**Figure 3:**
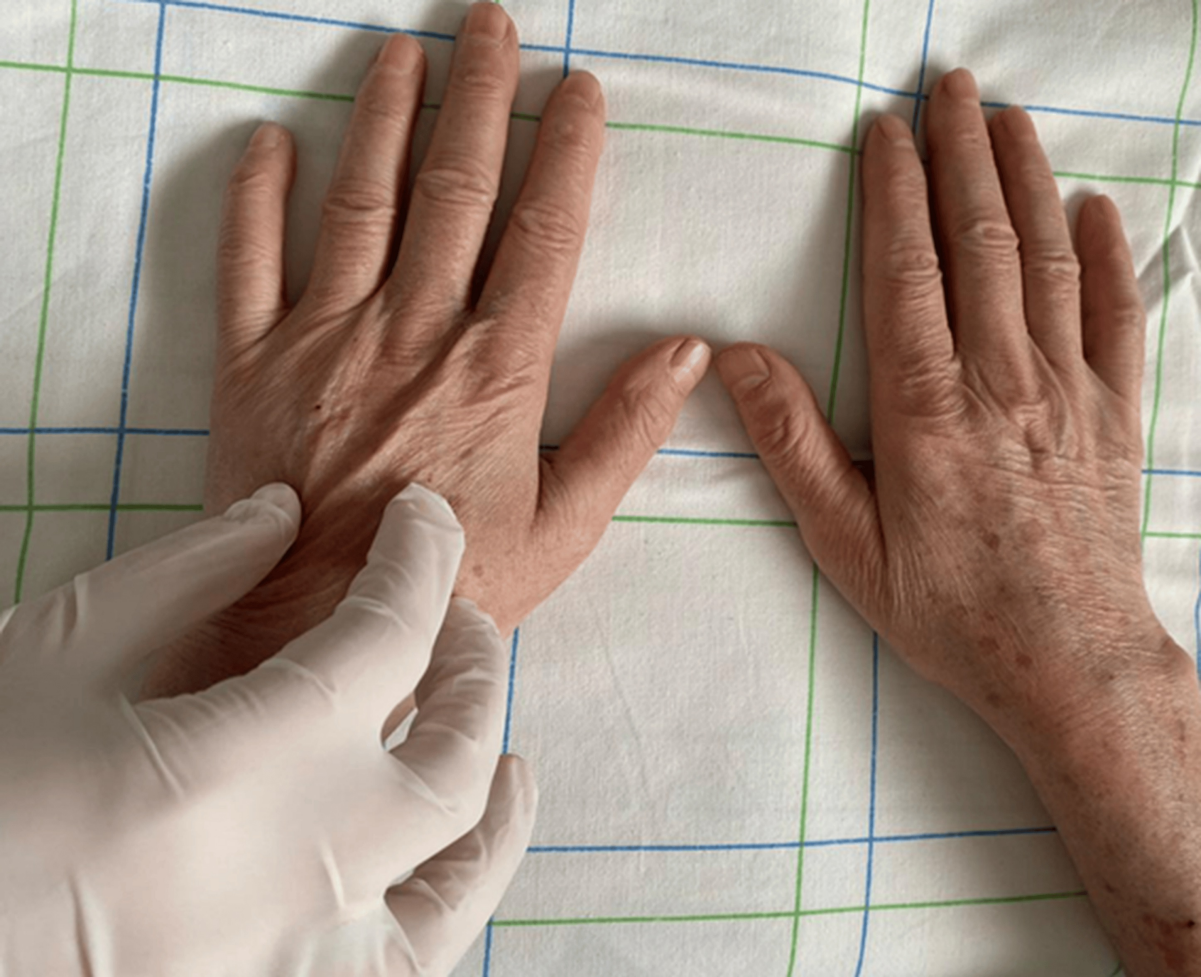
Cutaneous sclerosis spares the extremities

Clinical examination revealed generalized cutaneous sclerosis of the face (Figure 1), breast (Figure 2), as well as the upper and lower limbs, sparing the extremities (Figure 3). There was also limited mouth opening, with a maximum diameter of 2 cm. The doughnut sign was negative. Clinical examination of the digestive, pulmonary, cardiovascular, and neurological systems was normal. Ophthalmologic examination revealed bilateral cortico-nuclear cataracts.

**Figure 4:**
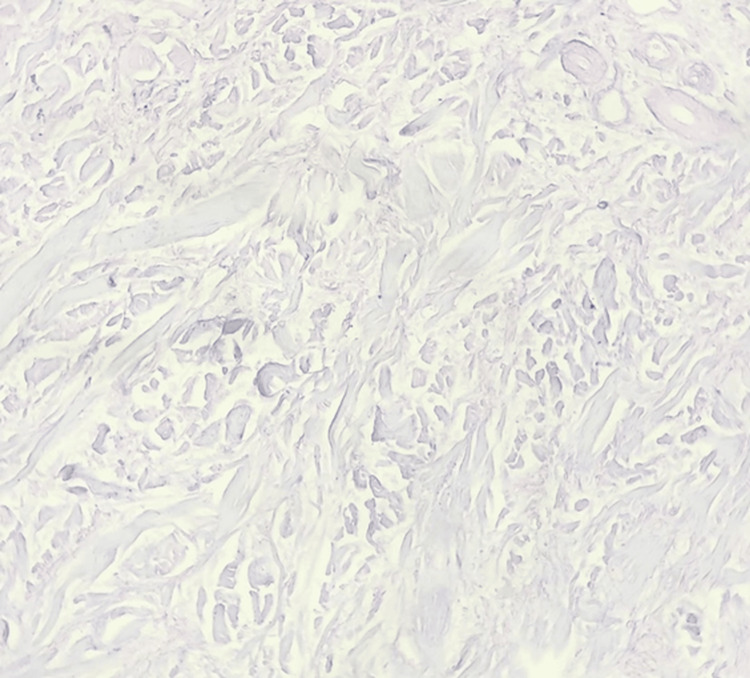
Alcian blue stain, showing the abundant deposits of mucin in the dermis between the collagen fibers (x400)

A skin biopsy revealed irregular fibrosis and proliferation of fibroblasts with interstitial mucin deposits. Alcian blue staining was positive in the upper and middle dermis (Figure 4). These histopathological findings confirmed the diagnosis of SM. 

Serum protein electrophoresis showed an inflammatory profile associated with double-wave gamma globulins with a narrow band. Additional immunofixation was requested, which showed the presence of monoclonal IgG kappa immunoglobulins. Quantification of serum free light chains showed an increase in Kappa-type free light chains to 51.54 mg/l (normal range: 3.30-19.40 mg/l). The Bence-Jones protein urine test revealed the absence of a band characteristic of Bence-Jones protein, which helped to exclude multiple myeloma in this case.

Myelogram showed the presence of 2% plasma cells (normal range: 1-3%) with a lack of blast cells or malignant cells. Bone marrow biopsy showed a lymphoid infiltrate of fairly moderate interstitial and nodular B phenotype; the exact nature of which required further investigation by flow cytometric immunophenotyping. Flow cytometric immunophenotyping revealed 10% (340 cells/µl) of CD19+ B cells (normal range: 100-500 cells/µl), 15 % (510 cells/µl) of CD5+B cells (normal range: 340-680 cells/µl), 62% (2108 cells/µl) of CD3+ T cells (normal range: 900-2000 cells/µl), 38% (1292 cells/µl) of CD4+ T cells (normal range: 500-1200 cells/µl), 24% (816 cells/µl) of CD8+ T cells (normal value: 150-1000 cells/µl). Altogether, these investigations excluded the presence of a malignant hematologic disorder.

**Figure 5:**
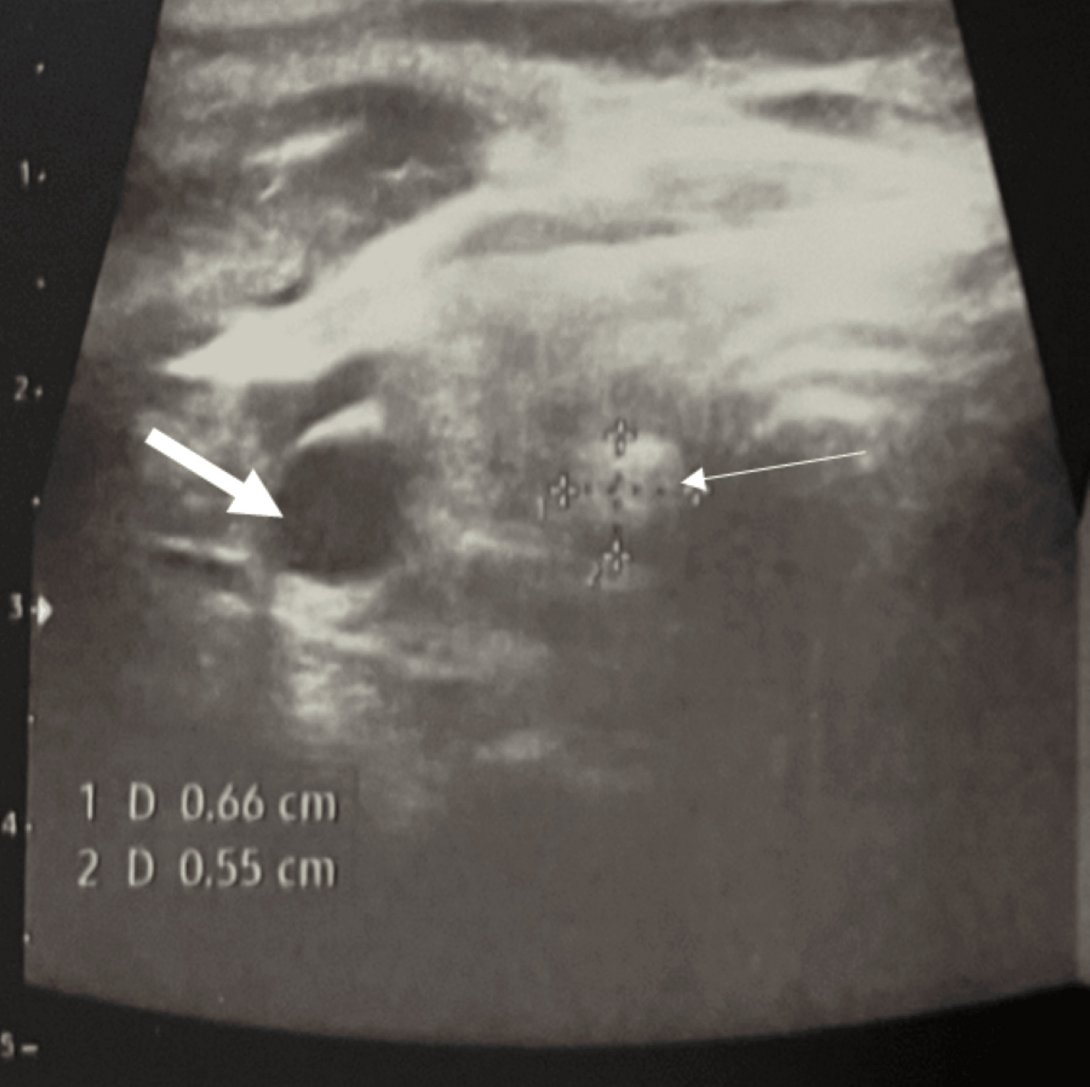
Thyroid ultrasound image showing an isthmic thyroid nodule (thin arrow) and a regenerative 'white knight' nodule (thick arrow) in the mid-portion of the right thyroid lobe

As for the thyroid work-up, the thyroid-stimulating hormone (TSH) level was 9.09 µU/ml (normal range: 0.4-4 µU/ml) with a free thyroxine (T4) level of 0.72 ng/dl (normal range: 0.7-1.8 ng/dl). Anti-TPO antibodies were 393.44 UI/ml (normal range: <5.61 UI/ml). A thyroid ultrasound was also performed and revealed an isthmic nodule, a well-defined oval lesion with cystic echotexture, measuring 6 x 2 mm, classified as Eutirads II, with two "white knight" nodules on a background of thyroiditis; one in the right middle lobe and the other in the left middle lobe (Figure 5). These findings are consistent with Hashimoto's autoimmune thyroiditis.

**Figure 6:**
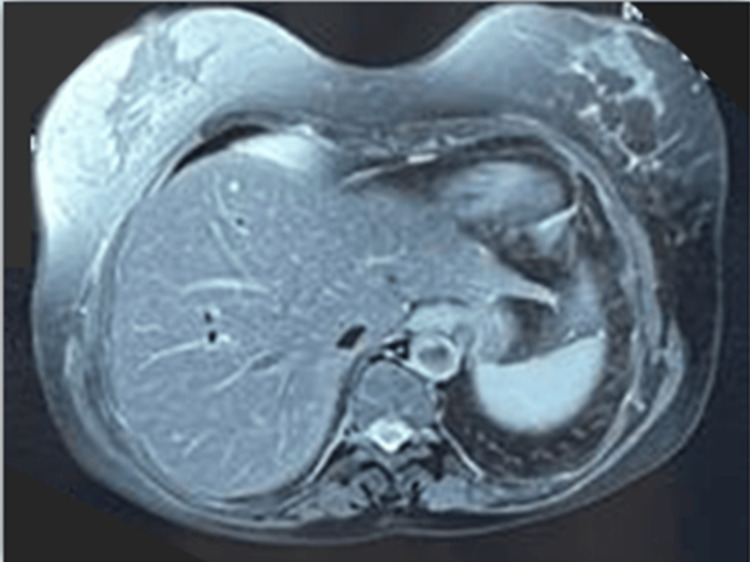
Magnetic resonance cholangiopancreatography (transverse section) at the hepatic level showing no dilatation of the common or intrahepatic bile ducts

Liver workup proved biological cholestasis without clinical signs; alkaline phosphatase (ALP) and gamma-glutamyl transferase (GGT) levels were 10 times the normal value. Abdominal ultrasound and magnetic resonance cholangiopancreatography (MRCP) showed a normal-sized liver, no dilatation of the intrahepatic bile ducts or the suprahepatic bile ducts, and a normal-sized portal trunk (Figure 6). An autoimmune liver test was carried out and found to be negative.

Liver biopsy was performed, which showed mild chronic hepatitis and came out with portal fibrosis without Metavir A1F1 score septa associated with destructive lymphocytic cholangitis in favor of primary biliary cholangitis, with absence of ductopenia or tumor lesion. Cardiac transthoracic ultrasonography was normal. Respiratory function tests showed a severe restrictive syndrome, but the examination was distorted by the lack of closure of the oral cavity during the test. Electroneuromyography proved normal nerve conduction in all four limbs. Esophagogastroduodenoscopy revealed an erythematous pangastritis. Gastric and intestinal biopsies were taken and showed a morphological appearance of discrete congestive gastropathy with the absence of gastric/intestinal metaplasia and dysplasia.

The patient received systemic corticosteroid therapy at a dose of 0.7 mg/kg/d for one month, then four cycles of intravenous human immunoglobulin (IVIG), at a dose of 2 g/kg spread over five days, per month, with no clinical improvement. The patient passed away five months after her last course.

## Discussion

According to the classification by Rongioletti and Rebora, lichen myxedematosus is divided into two forms: localized and generalized. The former is limited to the skin and does not present with monoclonal gammopathy. The latter form, also known as SM, is associated with monoclonal gammopathy and systemic symptoms. Atypical lichen myxedematosus is a generic term describing intermediate cases [4]. SM is also considered a primary cutaneous mucinosis generally associated with paraproteinemia.

Our case represents a rare association of SM, Hashimoto’s disease, and cholangitis, which has never been reported before. The cutaneous manifestations of SM are described as dense, waxy, slightly reddish or flesh-colored papules, dome-shaped or flat, 2-3 mm in diameter, non-pruritic, forming small aggregates, often arranged linearly. They mainly appear on the backs of the hands, the sides of the arms, the upper trunk or back, the face, or the axillae. The palms, scalp, and mucous membranes are spared [5]. As in our case, the sclerodermoid induration may precede the papules, the primary manifestation of SM. Two characteristic features may be observed: leonine facies, due to mucin deposition in the glabella and forehead that thickens skin folds; and the doughnut sign, due to thickening of the proximal interphalangeal joint with a central depression, especially pronounced during dorsal finger extension. In our case, these two features were absent. Complications may include joint contractures or stiffness of the facial muscles with difficulty opening the mouth. What distinguishes SM from systemic scleroderma is the presence of papular lesions in favor of SM, whereas telangiectasias and cutaneous calcinosis are suggestive of systemic scleroderma. Raynaud's phenomenon, typical of systemic scleroderma, is rare (approximately 10% of SM cases) [5].

SM may be complicated by systemic involvement that can be life-threatening: esophageal (dysphagia), pulmonary (obstructive or restrictive syndrome, pulmonary hypertension, emphysema), cardiac (pericardial effusion, acute coronary syndrome), renal (renal failure), articular (carpal tunnel syndrome, polyarthritis), ophthalmologic (corneal deposits, ectropion, submacular hemorrhages and macular edema), muscular (inflammatory myopathy), and neurological (encephalopathy, seizures, psychosis, stroke, peripheral neuropathy) [1,6,7]. In this case, there was digestive involvement: dysphagia to solids, while other systemic signs were absent.

For the positive diagnosis of SM, Rongioletti and Rebora proposed the following criteria in 2001 [4]: (i) A clinical examination of a generalized papular and sclerodermoid eruption, (ii) A cutaneous histological examination revealing mucin deposits in the reticular dermis, fibroblastic proliferation, and fibrosis, (iii) The presence of serum monoclonal gammopathy, and (iv) The absence of thyroid pathology.

An important differential diagnosis is generalized myxedema, which is seen in cases of long-term hypothyroidism and may present with cutaneous mucinosis and systemic symptoms that resemble SM. Hypothyroid myxedema is associated with generalized slowing of the body's metabolic processes and mucin deposit in various organs, including the skin, creating generalized edema. The clinical signs classically found most often are: macroglossia, periorbital puffiness, thick lips, and edema of the extremities. At the same time, the skin tends to be cold, dry, and pale, and the hair is rough, dry, and brittle, with diffuse partial alopecia. Histologically, we observe hyperkeratosis with follicular plugs and diffuse mucins as well as edema spreading between the collagen fibers extending over the entire dermis [8]. And unlike SM, there is no proliferation of fibroblasts.

With regard to the association of SM with hypothyroidism, as in our patient's case, a search of indexed articles was carried out on PubMed using the terms "scleromyxoedema, lichen myxoedematous, and hypothyroidism"; eight cases were found in the literature describing this association (Table 1). Four patients presented with monoclonal gammopathy, one IgG kappa monoclonal gammopathy, and three with IgG lambda monoclonal gammopathies. Patients with generalized papular eruptions associated with scleroderma and hypothyroidism without monoclonal gammopathy improved on thyroxine [9-12], whereas others who presented a clinical association of SM with hypothyroidism and monoclonal gammopathy improved on intravenous immunoglobulins [2,13,14].

**Table 1:** Summary of eight cases found in the literature describing the association between scleromyxoedema and hypothyroidism LM, lichen myxedematosus;  CNS, central nervous system;  IVIG, Intravenous immunoglobulin

Reference (year)	Clinical presentation	Skin histology	Monoclonal spike	Systemic Symptoms	Treatment	Outcome	Presumed Diagnosis
Archibald and Calvert, 1977 [13]	Recurrent transient ischemic attacks, grouped lichenoid papules on extremities	Mucin in upper dermis: fibroplasia	IgG κ chain	Asthenia	Thyroxine and Coumadin	Resolved CNS: unknown skin outcome	Atypical LM: possibly scleromyxoedema
Schaeffer et al., 1983 [7]	Hashimoto thyroiditis with cool dry skin, papules on extremities; no skin thickening	Diffuse mucin in lower dermis; no fibroplasia	No	None	UVB/thyroxine	Resolved	Atypical thyroid dermopathy
Martin-Ezquera et al., 2006 [9]	Papules on abdomen: subclinical hypothyroidism	Mucin: mild fibroplasia	No	None	Thyroxine	Resolved	Atypical thyroid dermopathy
Volpato et al., 2010 [10]	Large annular plaque; subclinical hypothyroidism	Inflammation, diffuse mucin; no fibroplasia	No	Fatigue, arthralgia	Thyroxine	Resolved systemic symptoms, skin persisted	Atypical LM
Macnab and Kenny, 2013 [11]	Generalized papular eruption; subclinical hypothyroidism	Mucin ; fibroplasia	IgG λ chain	CNS	Intravenous immunoglobulin	Resolved skin and CNS, thyroid uncontrolled	Scleromyxoedema
Shenoy et al., 2019 [12]	Progressive cutaneous thickening	Increased mucin, fibroblasts, and perivascular mixed inflammation	Delayed spike in IgG λ chain	None	Intravenous immunoglobulin	Cleared but recurred when dose was decreased	Scleromyxoedema
	Diffuse waxy papules	Palisading lymphocytes and histiocytes around mucin	None	Rheumatoid arthritis	Systemic steroids	Responded to systemic steroids but recurred with cessation of therapy	LM
Hazan et al. 2020 [2]	Progressive cutaneous thickening and papules ranged from flesh colored to erythematous	Increased fibroblasts and increased mucin accumulation in the dermis	IgG λ chain	Acute concerns of confusion and muscle weakness	intravenous levothyroxine, systemic steroids, and a course of intravenous immunoglobulin (IVIG) + lenalidomide 25 mg/d	Resolved skin but thyroid is uncontrolled	SM

Several therapeutic options are available for the treatment of SM. First-line treatment relies on IVIG, with high response rates: 69% for cutaneous involvement, 75% for paraproteinemia, and 63% for systemic manifestations [15,16]. IVIG is typically administered at a dose of 2 g/kg over a period of two to five consecutive days per month. Infusions are initially scheduled at four-week intervals. Discontinuation of therapy often leads to relapse, but symptoms usually respond effectively to re-initiation of the same treatment. Maintenance cycles every six to eight weeks are generally required to sustain remission. A therapeutic response should be expected within six months; if no response is observed after this period, treatment should be discontinued [17].

In the event of IVIG failure, disease progression, or treatment intolerance, thalidomide or systemic corticosteroids can be administered as second-line therapy. Thalidomide acts on multiple disease manifestations, with high response rates for cutaneous lesions (69%), paraproteinemia (69%), and systemic symptoms (80%) [18-20]. The initial dose of thalidomide is 50-100 mg per day, which can be gradually increased up to 150-400 mg per day depending on clinical condition and tolerance [17]. Lenalidomide, a thalidomide analogue with a better toxicity profile, may also yield satisfactory results. It is administered at a dose of 25 mg per day for three weeks per month. Due to its immunomodulatory properties, it may help maintain long-term remission after discontinuation of IVIG [17].

Corticosteroids are used in SM either as monotherapy or in combination with other treatments. This approach has shown high response rates: 72.7% for cutaneous involvement, 100% for systemic symptoms, and 75% for paraproteinemia. Prednisone (0.5-1 mg/kg/day), prednisolone (0.3-0.5 mg/kg/day), and high-dose oral dexamethasone (40 mg once daily, four days per week for three consecutive weeks each month) can effectively target both immunoglobulin production and fibroblast hyperactivity due to their immunosuppressive and antifibrotic effects [17]. In severe or refractory cases, melphalan or bortezomib may be proposed as third-line therapy [17].

## Conclusions

Although classically considered an exclusion criterion, the association between SM and hypothyroidism, particularly Hashimoto's thyroiditis, has been reported in rare cases, as illustrated by our observation. This co-occurrence challenges the strict diagnostic criteria and highlights the importance of a thorough clinical and paraclinical evaluation of all patients presenting with a suggestive clinical presentation. Given the extreme rarity of this association and the patient’s death after four cycles of intravenous immunoglobulins, long-term follow-up or comparison with similar cases was not feasible.
